# antiSMASH 5.0: updates to the secondary metabolite genome mining pipeline

**DOI:** 10.1093/nar/gkz310

**Published:** 2019-04-29

**Authors:** Kai Blin, Simon Shaw, Kat Steinke, Rasmus Villebro, Nadine Ziemert, Sang Yup Lee, Marnix H Medema, Tilmann Weber

**Affiliations:** The Novo Nordisk Foundation Center for Biosustainability, Technical University of Denmark, Kemitorvet bygning 220, 2800 Kgs. Lyngby, Denmark; The Novo Nordisk Foundation Center for Biosustainability, Technical University of Denmark, Kemitorvet bygning 220, 2800 Kgs. Lyngby, Denmark; German Centre for Infection Research (DZIF), Interfaculty Institute of Microbiology and Infection Medicine, Auf der Morgenstelle 28, University of Tübingen, 72076 Tübingen, DE, Germany; The Novo Nordisk Foundation Center for Biosustainability, Technical University of Denmark, Kemitorvet bygning 220, 2800 Kgs. Lyngby, Denmark; German Centre for Infection Research (DZIF), Interfaculty Institute of Microbiology and Infection Medicine, Auf der Morgenstelle 28, University of Tübingen, 72076 Tübingen, DE, Germany; The Novo Nordisk Foundation Center for Biosustainability, Technical University of Denmark, Kemitorvet bygning 220, 2800 Kgs. Lyngby, Denmark; Department of Chemical and Biomolecular Engineering (BK21 Plus Program) and BioInformatics Research Center, Korea Advanced Institute of Science and Technology, 291 Daehak-ro, Yuseong-gu, Daejeon 34141, South Korea; Bioinformatics Group, Wageningen University, Droevendaalsesteeg 1, 6708PB Wageningen, the Netherlands; The Novo Nordisk Foundation Center for Biosustainability, Technical University of Denmark, Kemitorvet bygning 220, 2800 Kgs. Lyngby, Denmark

## Abstract

Secondary metabolites produced by bacteria and fungi are an important source of antimicrobials and other bioactive compounds. In recent years, genome mining has seen broad applications in identifying and characterizing new compounds as well as in metabolic engineering. Since 2011, the ‘antibiotics and secondary metabolite analysis shell—antiSMASH’ (https://antismash.secondarymetabolites.org) has assisted researchers in this, both as a web server and a standalone tool. It has established itself as the most widely used tool for identifying and analysing biosynthetic gene clusters (BGCs) in bacterial and fungal genome sequences. Here, we present an entirely redesigned and extended version 5 of antiSMASH. antiSMASH 5 adds detection rules for clusters encoding the biosynthesis of acyl-amino acids, β-lactones, fungal RiPPs, RaS-RiPPs, polybrominated diphenyl ethers, C-nucleosides, PPY-like ketones and lipolanthines. For type II polyketide synthase-encoding gene clusters, antiSMASH 5 now offers more detailed predictions. The HTML output visualization has been redesigned to improve the navigation and visual representation of annotations. We have again improved the runtime of analysis steps, making it possible to deliver comprehensive annotations for bacterial genomes within a few minutes. A new output file in the standard JavaScript object notation (JSON) format is aimed at downstream tools that process antiSMASH results programmatically.

## INTRODUCTION

Bacterial and fungal natural products constitute a key source of scaffolds for the development of antimicrobials and other drugs (1), and mediate ecological interactions between organisms in various ways (2).

Mining genomic data for the presence of biosynthetic pathways that enable organisms to produce such molecules, which are also referred to as secondary or specialized metabolites, have become an essential approach that complements activity- and chemistry-guided isolation and identification approaches (3). Several computational tools, such as CLUSEAN (4) or PRISM (5), have been developed to support scientists with this task. The ‘antibiotics and secondary metabolites analysis shell’, antiSMASH, is a pioneer amongst these tools. Initially released in 2011 (6), it has since been further extended and improved (7–12), and is currently used by thousands of academic and industrial scientists worldwide to identify so called secondary metabolite ‘biosynthetic gene clusters’ (BGCs) in their genomes of interest. In 2017, a database component was added to the antiSMASH framework, which provides instant access to thousands of pre-computed antiSMASH genome mining results of publicly available genomes (13,14). Furthermore, several independent tools, such as the mass-spectrometry guided peptide mining tool Pep2Path (15), the ‘Antibiotic Resistance Target Seeker’ ARTS (16), the sgRNA design tool CRISPy-web (17), a reverse-tailoring tool to match finished NRPS/PKS structures to antiSMASH-predicted core structures (18) and the BGC clustering and classification platform BiG-SCAPE (19) were developed that directly interact with and interpret results generated by antiSMASH and provide information that is outside the scope of a core-antiSMASH analysis.

Here, we present version 5 of antiSMASH, which contains many improvements. In addition to many features visible to the end users, such as extended and improved BGC detection and analysis capabilities and a modernized and improved User Interface (see below), antiSMASH version 5 was completely rewritten in Python version 3 and the code was restructured to increase performance, reliability and ease of maintenance. This has led to a significant speed increase of the pipeline. A complete list of antiSMASH 5 features is included in the antiSMASH documentation https://docs.antismash.secondarymetabolites.org/antiSMASH5features/.

## NEW FEATURES AND UPDATES

### New gene cluster classes and refinement of cluster detection rules

The most widely used and recommended mode to detect BGCs in genomic data is via manually curated and validated gene cluster rules. These are based on identifying co-occurring conserved core enzymes in the genome using HMM-profiles that were derived from Pfam (20), SMART (21), BAGEL (22) or Yadav *et al.* (23), or that were created specifically for antiSMASH. While antiSMASH version 4 supported the rule-based detection of 44 different biosynthetic types, antiSMASH 5 now includes rules for 52 different BGC types. In version 5, new rules were added to detect BGCs encoding the biosynthesis of N-acyl amino acids (24), β-lactones (25), polybrominated diphenyl ethers (26), C-nucleosides (27), pseudopyronines (28), fungal RiPPs (29–31) and RaS-RiPPs (32,33). Furthermore, a new ‘nrps-like’ rule was defined for NRPS-fragments, i.e. atypical NRPSs that don’t have the typical C-A-T module architecture. The previous ‘otherks’ rule was split into two rules to individually assign heterocyst glycolipid synthase-like clusters and other atypical PKSs. In addition, some rules were improved based on user case reports. The rules describing lanthipeptides and trans-AT type I PKS were refined to reduce the number of false positive hybrid calls on other cluster types. For trans-AT- type I PKS and type II PKS, we increased the size of the cluster cutoffs to capture previously missed tailoring enzymes in published clusters. The rule for linear azole/azoline-containing peptides was made more generic to better cover the range of described clusters.

The rule describing microcin clusters was removed, as microcins are a class of RiPPs defined via their production in *Enterobacteriaceae*, and are already captured by one of our other specific RiPP cluster rules, depending on their respective biosynthesis pathway (e.g. microcin J25-like RiPPs were previously covered by the old microcin cluster rules but chemically are lasso peptides, while microcin B17 is a linear azol(in)e-containing peptide).

### Improved type II PKS prediction

Bacterial type II PKS BGCs code for the biosynthesis of aromatic polyketides, such as the antibiotic tetracycline or the anti-tumour drug doxorubicin. From the beginning, antiSMASH has had rules that were able to detect type II PKS BGCs by checking for the presence of the KS*α* and KS*β*/CLF component of the minimal PKS. However, no detailed prediction methods had been added since antiSMASH’s first version. In antiSMASH 5, we introduce a new PKS II analysis module (12), which uses a collection of manually curated HMMs to predict potential starter units, the number of elongation cycles (and thus a rough estimation of the putative molecular weight of the core compound), cyclization patterns and some conserved type II PKS specific tailoring reactions. This module is automatically triggered whenever a type II PKS BGC is detected.

### Annotation of resistance genes via Resfams

The Resfams database (34) is a curated database of protein families with confirmed antibiotic resistance function. antiSMASH 5 uses the profile Hidden Markov Models (pHMMs) from Resfams to annotate potential resistance genes found in predicted gene regions. Potential resistance gene-hits are displayed in the ‘gene details’ panel along with other functional annotations.

### GO-term annotations

The Gene Ontology (GO) is a controlled vocabulary for describing biological processes, molecular functions and cellular components in a consistent way to enable comparison of these between different species. Amongst its wide range of uses, the GO has been used to predict gene clusters in eukaryotes and bacteria (35) and, in conjunction with antiSMASH, to refine cluster boundaries in antiSMASH output for *Aspergillus* species (36).

To facilitate these and other GO-based analyses, antiSMASH 5 includes an option to automatically annotate GO terms on Pfam domains. This functionality makes use of the fact that GO terms may be linked not only to specific gene products, but also to other means of classification in so-called ‘mappings’ (http://geneontology.org/page/download-mappings). As antiSMASH can automatically annotate Pfam domains, the GO annotation functionality makes use of the Pfam to GO mapping supplied by the Gene Ontology Consortium’s website (37). If the ID of a predicted Pfam domain in an antiSMASH record is present in the Pfam to GO mapping, the respective GO terms are assigned and presented in the ‘gene details’ panel.

### Link to the antiSMASH database

antiSMASH provides options to search for similar gene clusters in public datasets. As already implemented in previous versions of the software, the KnownClusterBlast functionality searches each identified region against the manually curated MIBiG (38) repository. The KnownClusterBlast and ClusterBlast search functions use an algorithm first described in antiSMASH 1 (6), which also is in use in a generalized version in MultiGeneBlast (39). In the previous versions of antiSMASH, the ClusterBlast database was generated by scripts that used the antiSMASH BGC detection logic on sequences downloaded from the NCBI Genbank/RefSeq databases. As version 2 of the antiSMASH database now also contains BGCs of draft genomes (14), starting with antiSMASH 5 the ClusterBlast databases will be directly generated from the new antiSMASH database and complemented with individual BGC records that were submitted to NCBI outside of whole-genome submissions. This provides several advantages: The abundance of entries for selected genera/species in the public databases (and thus also in the previous ClusterBlast database) is strongly skewed towards clinically or industrially relevant organisms. There are, for example, more than 15 000 assemblies for *Escherichia coli* deposited at NCBI. For the antiSMASH database, a sequence-based dereplication workflow was established (14) that reduced the number of redundant entries with very high sequence similarity. Thus, the updated ClusterBlast database contains fewer entries than the previous release, despite the increase in publicly available sequence data. This decrease has resulted in reduced computation times, while simultaneously providing more relevant hits. Furthermore, as the entries of the ClusterBlast database are directly related to the BGCs in the antiSMASH database, a link to the respective BGC is now included for all ClusterBlast hits, promptly directing the user to the detailed report of the similar gene clusters.

### New ‘*region*’ concept

In previous versions, antiSMASH referred to all co-located, hybrid and independent BGCs with the single label ‘cluster’. In many cases, this led to confusing structure predictions when distinct BGCs are encoded side-by-side. For example, many *Streptomyces* plasmids exist for which all BGCs lie so close to each other that all were joined into a single large ‘cluster’. In order to better distinguish the different biological options that lead to BGCs, antiSMASH 5 introduces some new terminology.

The definitions now used in antiSMASH 5 are:

*Core*: The minimum area containing one or more genes that code for enzymes for a single BGC type that are detected by the manually curated detection rules. These genes do not have to be contiguous, but can be within a certain cutoff distance as defined by the detection rule for the BGC type in question.

*Neighbourhood*: Distance up- and downstream of the cluster *core* that is used to find tailoring genes/enzymes; the neighbourhood distances for the individual biosynthetic types were empirically determined and defined in the detection rules.

*Protocluster*: Contains *core* + *neighbourhoods* at both sides of the *core*; each *protocluster* always will have one single product type (for example, NRPS). *Protoclusters* may overlap partially or completely with other *protoclusters*. In the result webpage, protoclusters are displayed as boxes above the gene arrows. The *cores* are shown as solid colour boxes, the *neighbourhoods* are the half-transparent areas around the cores.

*Candidate cluster*: Contains one or more *protoclusters*; the *candidate clusters* are defined as described below. These definitions better allow modelling of hybrid clusters, such as PKS/NRPS hybrids, which combine two or more different biosynthetic classes (as identified in the detection rules), or cases where one class is used to biosynthesize a precursor for a second class. An example of the latter is found in glycopeptide biosynthesis, where one of the amino acids is synthesized by a type III PKS, which is then incorporated into the product by a NRPS. *Candidate clusters* may overlap partially or completely with other *candidate clusters*. In the result webpage, *candidate clusters* are shown as boxes above the *protoclusters*.

*Region*: Contains one or more *candidate clusters*; The *regions* in antiSMASH 5 correspond to the entities called ‘clusters’ in antiSMASH 1 – 4 and now constitute what is displayed on a page of the results webpage. Sometimes, a region will contain multiple mutually exclusive *candidate clusters*; in such cases, comparative genomic analysis and/or experimental work is required to assess which of these candidate clusters constitute actual BGCs. *Regions* will not overlap with each other. At least one of the contained *candidate clusters* will cover the full length of the *region*.

There are four kinds of candidate clusters: *chemical hybrids, interleaved, neighbouring* and *single*.

**Figure 1.**
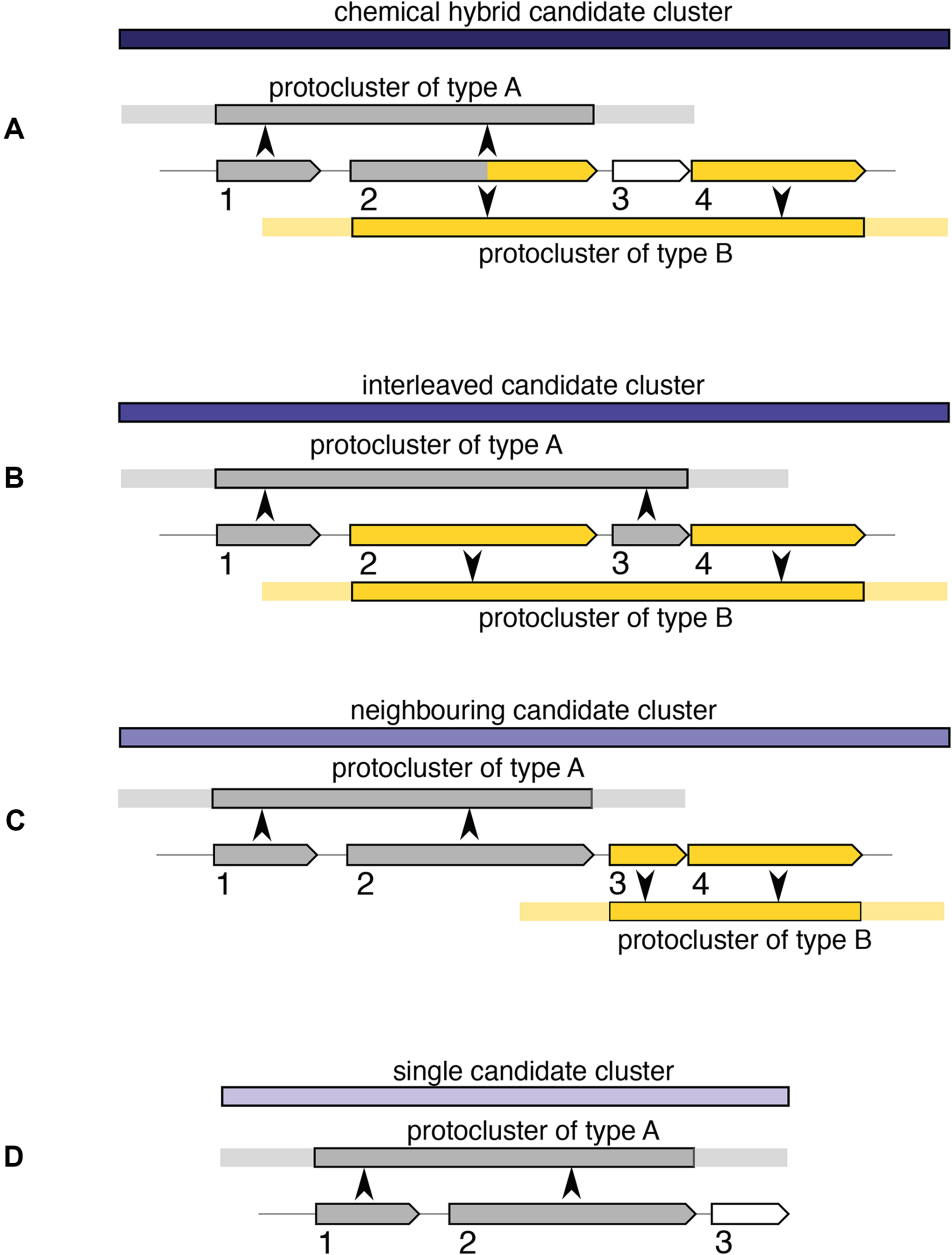
Candidate cluster types. 1,2,3,4: Grey/yellow: gene involved in *protocluster* A/B. (**A**) Chemical Hybrids. Since cluster type A and cluster type B share a CDS that defines those *protoclusters*, they are classified as ‘chemical hybrid’. (**B**) Interleaved: Since none of the *protoclusters* share any defining CDS with any other *protocluster*, it is not annotated as a *chemical hybrid*, even though the biosynthetic product may or may not be. The two *protoclusters* form an *interleaved candidate clusters*, since the core of A overlaps with the core of B. (**C**) Neighbouring: *Neighbouring candidate clusters* are defined if the neighbourhoods of two *protoclusters* but not their cores overlap. (**D**) Singles: If *protoclusters* don’t have any overlap/relation with other *protoclusters*, the term *single candidate cluster* is assigned.

*Chemical hybrid candidate clusters* contain at least two *protoclusters* that share at least one gene that codes for enzymes of two or more separate BGC types (e.g. a single gene coding for type I PKS and NRPS modules) (Figure 1A). An example of this type are hybrid PKS/NRPSs. Please note that this type of *candidate cluster* can also include *protoclusters* within that shared range that do not share a coding sequence provided that they are completely contained within the *candidate cluster*.

*Interleaved candidate clusters* contain *protoclusters* that do not share cluster-type-defining coding sequences, but their *core* locations overlap (Figure 1B).

*Neighbouring candidate clusters* contain *protoclusters* which transitively overlap in their neighbourhoods (Figure 1C).

*Single candidate clusters* (Figure 1D) exist for consistency of access, they contain only a single *protocluster*. Note that individual *protoclusters* can be contained by more than one *candidate cluster* (typically a *neighbouring candidate cluster* and one of *single, interleaved or chemical hybrid*).

Each *candidate cluster* assignment is transitive, for example if a *protocluster* would form a *chemical hybrid* with each of two neighbouring *protoclusters*, but these neighbours would not form a *chemical hybrid* on their own, all three together will still form a *chemical hybrid candidate cluster*.

### Improved user interface

A central aim of antiSMASH is to provide very detailed and specific information via an easy to use and understand user interface (UI). The UI remained principally unchanged from the initial release of antiSMASH in 2011, despite the increased functionality added with each new version. In this version, we have modernized the UI using updated web technologies that allow a better structuring of the result-content of the antiSMASH results pages. For redesigning the UI, it was important that the reliable and well-established look-and-feel was conserved, while also retaining the ability to download the whole web-based results folder and to display it locally in a variety of web-browsers.

We and others (such as (40)) have realized that antiSMASH results using the heuristic ClusterFinder algorithm (41) were, more often than not, wrongly interpreted. At the same time, ClusterFinder contributed significantly to the computational workload. For these reasons, we decided to remove this feature from the pubic antiSMASH web server. It is, of course, still included in the download version of antiSMASH and can be enabled via the command line.

**Figure 2.**
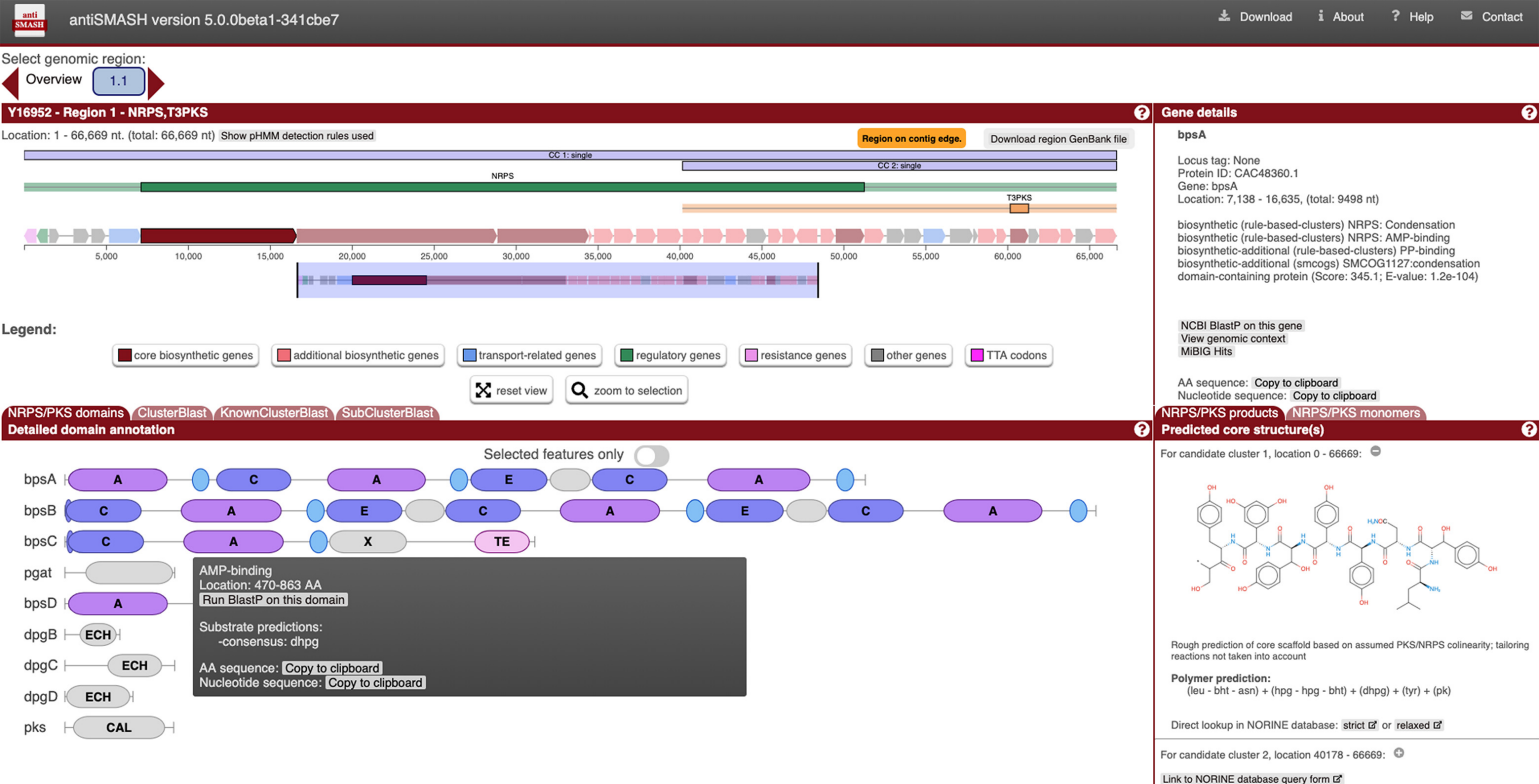
Screenshot of the antiSMASH 5 user interface (example: NCBI-acc: Y16952; balhimycin BGC). The new region overview now allows panning/zooming. The *candidate cluster* and *protocluster* boxes are explained in the ‘new region concept’ section above. Information about the currently selected gene are displayed at the right ‘Gene details’ panel. For PKS or NRPS regions, the detailed domain annotation is displayed; by pressing the tabs, users can select the domain overview (shown) or the ClusterBlast, KnownClusterBlast or SubClusterBlast results. At the right, the structure prediction and details of specificity predictions are displayed upon selecting the plus sign.

In the Regions overview section (Figure 2), a graphical overview showing the location of the identified regions on the chromosome/plasmid/scaffolds/contig is displayed. In the detailed view, regions that are located on contig-borders are now clearly labelled. This often indicates that parts of the BGC are missing or that several sections of a BGC are located on different contigs and are therefore reported individually (for a more detailed discussion on this phenomenon, please see (42)). For the first time, antiSMASH 5 now offers interactive browsing of the BGCs, including selection of ‘functional’ units, i.e. core enzymes, transporters, etc., zooming to individual genes or *regions/candidate clusters/protoclusters*. Details of the selection are now provided in side panels instead of pop-up windows, using a hierarchical view of the analysis summaries (which can be expanded by clicking ‘+’) to provide additional details. For the display of the PKS/NRPS domain organization, the user now can choose whether to limit the shown domains to the currently selected genes or just display the results of the selected gene(s)/enzyme(s). Furthermore, the information is now organized in ‘tabs’ that do not require scrolling down along an often very long results page.

## CODE REFACTORING AND SPEED-UP

Large parts of the pre-antiSMASH 5 code base were still derived from antiSMASH version 1, which was released in 2011. In order to maintain future compatibility, the antiSMASH code base had to be migrated from python 2.7, which will reach end-of-life in 2020, to the current versions 3.5–3.7. As this transition required significant modification to the antiSMASH code, we decided to take this as a chance to completely rewrite the software with a special consideration on runtime, code stability and code maintainability. A unit test and integration test framework was implemented that covers most parts of the antiSMASH 5 code allowing a much easier debugging and—most importantly—extension of the code while at the same time ensuring that new features do not negatively impact the results of existing modules. For some of the externally contributed modules (Sandpuma, trans-AT PKS comparisons, terpene PrediCAT), our contributors are currently preparing updated and compliant versions, which will be added to antiSMASH 5 in minor releases once they are finished and tested. Like the earlier antiSMASH versions, antiSMASH 5 provides the analysis results in an interactive webpage and richly annotated GenBank-format files for the whole genome and individual clusters. As a new feature in version 5, all data are also available as a computer readable JSON container, which allows third party tools to easily process antiSMASH annotations. This JSON output has superseded some other output types, such as BioSynML and XLS.

In addition to the advantages mentioned above, the code refactoring and cleanup has also led to a significant speed increase of the new version by a factor of 4-11× (depending on genome and selected options); instead of waiting times of several hours, antiSMASH results are now usually delivered within 30–40 min after the start of the job for a typical submission at the public web server.

## CONCLUSIONS AND FUTURE PERSPECTIVES

With the help of software like antiSMASH, genome mining for specialized metabolites has established itself as a complementary approach for the identification of novel metabolites, which is routinely used within the natural products research community and increasingly applied in related fields such as metagenomics, environmental biology or metabolic engineering. With the improvements to the antiSMASH user interface and performance, we keep pace with these developments. Furthermore, the complete refactoring of the antiSMASH 5 code base will allow us to increasingly use antiSMASH as a tool that provides analysis data on which other software can perform additional analyses.

## DATA AVAILABILITY

antiSMASH is available from https://antismash.secondarymetabolites.org/ (bacterial version) or https://fungismash.secondarymetabolites.org/ (fungal version). The antiSMASH documentation, including a PDF user guide, is available from https://docs.antismash.secondarymetabolites.org. These websites are free and open to all users and there is no login requirement. The antiSMASH source code is available from https://github.com/antismash/antismash. antiSMASH is also available via Docker.
